# Social Prescribing Programmes to Prevent or Delay Frailty in Community-Dwelling Older Adults

**DOI:** 10.3390/geriatrics4040065

**Published:** 2019-11-27

**Authors:** Toby O Smith, Oluseyi F Jimoh, Jane Cross, Louise Allan, Anne Corbett, Euan Sadler, Mizanur Khondoker, Jennifer Whitty, Jose M Valderas, Christopher Fox

**Affiliations:** 1 Nuffield Department of Orthopaedics, Rheumatology and Musculoskeletal Sciences, University of Oxford, Oxford OX3 7LD, UK; 2 Faculty of Medicine and Health Sciences, University of East Anglia, Norwich NR4 7TJ, UK; O.Jimoh@uea.ac.uk (O.F.J.); J.Cross@uea.ac.uk (J.C.); M.Khondoker@uea.ac.uk (M.K.); Jennifer.Whitty@uea.ac.uk (J.W.); Chris.Fox@uea.ac.uk (C.F.); 3 College of Medicine and Health, University of Exeter, Exeter EX4 4QJ, UK; L.Allan@exeter.ac.uk (L.A.); A.M.J.Corbett@exeter.ac.uk (A.C.); J.M.Valderas@exeter.ac.uk (J.M.V.); 4 School of Health Sciences, University of Southampton, Southampton SO16 7PP, UK; E.A.Sadler@soton.ac.uk

**Keywords:** social prescribing, community referral, referral schemes, well-being programmes, older adults

## Abstract

The increasing incidence of frailty is a health and social care challenge. Social prescription is advocated as an important approach to allow health professionals to link patients with sources of support in the community. This study aimed to determine the current evidence on the effectiveness of social prescribing programmes, to delay or reduce frailty in frail older adults living in the community. A systematic literature review of published (DARE, Cochrane Database of Systematic Reviews, MEDLINE, EMBASE, CINAHL, NICE and SCIE, National Health Service (NHS) Economic Evaluation Database) and unpublished databases (OpenGrey; WHO Clinical Trial Registry; ClinicalTrials.gov) were searched to July 2019. Studies were eligible if they reported health, social or economic outcomes on social prescribing, community referral, referral schemes, wellbeing programmes or interventions when a non-health link worker was the intervention provider, to people who are frail living in the community. We screened 1079 unique studies for eligibility. No papers were eligible. There is therefore a paucity of evidence reporting the effectiveness of social prescribing programmes for frail older adults living in the community. Given that frailty is a clinical priority and social prescribing is considered a key future direction in the provision of community care, this is a major limitation.

## 1. Introduction

Frailty has been defined as a state of reduced resilience and increased vulnerability to adverse outcomes such as falls, disability, functional dependency, hospitalisation and death [1]. Interventions based on modifying physical activity participation, nutritional input and psychosocial engagement have been previously shown to delay or prevent frailty in older people, when prescribed by a healthcare provider [2,3].

Social prescribing via United Kingdom (UK) primary care services, allows health professionals to link patients with sources of non-medical support in the community, often provided by a third sector (including charities, voluntary, and community groups) [4]. Through this, community services can signpost interventions, provide information, make community referrals or instigate care navigation and coordination to a variety of non-National Health Service (NHS) services such as exercise groups, day centres, charitable organisations offering psychological therapies or nutritional-dietary groups. This is purported to provide a more sustainable and flexible model for long-term community care [5]. It also offers a model of care which is highly personalised, and therefore may be valuable particularly for older people who have heterogeneous health challenges.

The UK Department of Health and Social Care proposed social prescription for individuals with chronic care needs including frailty [6]. No systematic review has addressed the effectiveness of social prescribing for the prevention or management of frailty. Given the current promotion of social prescribing for this population, understanding the evidence-base underpinning this recommendation is paramount.

The purpose of this systematic review was to determine the current evidence on the effectiveness of social prescribing programmes to delay or reduce frailty in frail older adults living in the community.

## 2. Materials and Methods

This systematic review protocol was published on PROSPERO (Reference: CRD42019141868). This report adheres to the PRISMA reporting guideline [7].

### 2.1. Search Strategy

An electronic search of published databases (DARE, Cochrane Database of Systematic Reviews, MEDLINE (Ovid) Embase, PubMed, CINAHL, NICE and SCIE), NHS Economic Evaluation Database (NHS EED) and unpublished databases (OpenGrey, the WHO Clinical Trial Registry and ClinicalTrials.gov) were searched for eligible studies. The MEDLINE search is presented as Supplementary File S1. This was modified for each database.

Evaluation reports of social prescribing projects in the UK were searched using Google search, Google Scholar and websites of specific organisations, e.g., NHS Evidence, Kings Fund, Health foundation, Nuffield Trust and NESTA. Finally, the reference lists of all potentially eligible studies, review papers or project reports were reviewed. Databases were reviewed from inception to 1 July 2019.

### 2.2. Eligibility Criteria

Participants of interest were community-dwelling people aged 65 years and above, identified as frail or prefrail. We accepted any criteria used to diagnose frailty. Studies were eligible if they reported health, social or economic outcomes on social prescribing, community referral, referral schemes, wellbeing programmes or interventions where a non-health link worker was the intervention provider. Interventions of interest included:

1. Any physical activity programmes such as: exercise, dance, gym-based activities, guided or health walks, swimming or aqua therapy, team sports and cycling.
2. Any nutrition intervention including but not limited to: diet clubs, food clubs, cooking clubs, cooking courses, lunch clubs, weight management and diet therapy.
3. Psychosocial support such as: social support groups, psychoeducation, physical activity, peer-support groups, befriending, day clubs and social skill training.

We excluded participants who were prescribed physical activity, nutrition or psychosocial support as part of a health service prescription, and not a social prescription. We excluded any reports not presented in English.

The primary outcome measure was a validated measure of frailty. Secondary outcomes included: functional outcomes, body mass index, psychosocial wellbeing, social support assessed using a validated outcome, social participation, health-related quality of life (HRQoL), carer burden, mortality and health resource use, including hospital admission and direct and indirect health and societal costs. 

### 2.3. Study Identification

The search results were independently reviewed by two researchers (O.J., T.S.). Any discrepancies were resolved through discussion. No eligible papers were identified. There were no studies from which data was able to be extracted, appraised or analysed. Further details on the pre-planned methods are presented in the systematic review protocol.

## 3. Results

**Figure 1:**
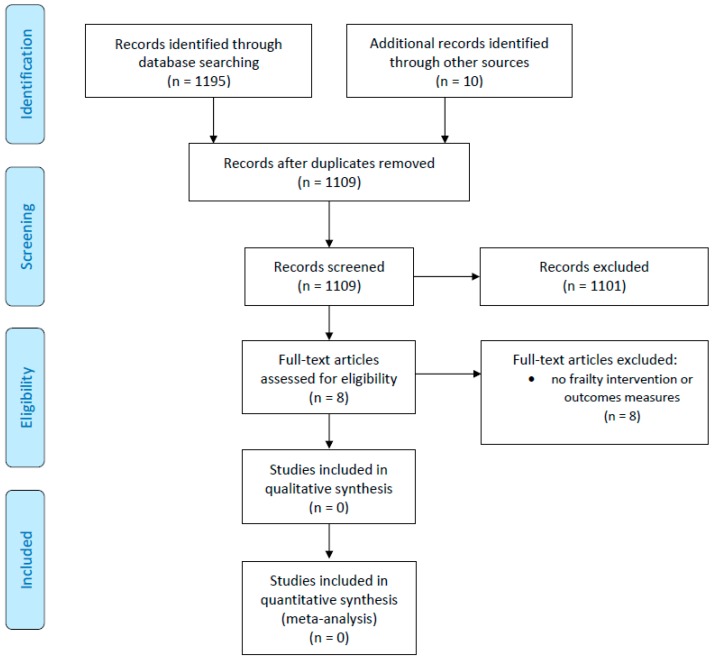
PRISMA flow-chart summarising the systematic review search results.

The results of the search strategy are presented in Figure 1. In total, 1079 unique studies were screened for eligibility after removing duplicates. Of these, eight were assessed for eligibility in a full-text review. No papers or reports were eligible. The principal reason for ineligibility was that no frailty measures was recorded before and after the intervention. Accordingly, there was no data on changes in frailty status. Secondly, interventions were offered not because participants were frail, but for other reasons such as the need to keep fit, ongoing health issues like diabetes, cardiovascular disease and other comorbidities. 

## 4. Discussion

These findings confirm a paucity of evidence reporting the effectiveness of social prescribing programmes for frail older adults living in the community. Given that frailty is a clinical priority for the NHS, with social prescribing considered a key future direction in the provision of community care [6], this is a major limitation. Understanding how to deliver this is a research priority.

Whilst previous systematic reviews have been undertaken on social prescribing [8,9], none have identified studies reporting the results of social prescribing interventions for frail older adults. It is not possible to extrapolate the findings of interventions such as physical activity, nutrition and psychosocial strategies delivered by healthcare services to a social prescription model. There are innate differences in the methods of delivery, most notably in staffing and locations of delivery. There may also be differences in uptake of social prescribing interventions compared to those delivered by NHS and other ‘conventional’ healthcare providers for people with long-term conditions [10]. Acknowledging these differences and understanding the decision-making of service-users, carers and health and social care professionals will aid in the development of successful social prescribing interventions for implementation in community care.

Although not eligible, as the study did not specifically recruit people who were frail or pre-frail, Elston et al. [11] reported clinical outcomes of a social prescribing intervention for 86 older people in one UK healthcare trust. The intervention was the provision of a ‘link-worker’ for 12 weeks for older individuals to identify goals and was sign-posted to community and voluntary sector groups. They reported benefits in clinical outcomes and social participation, but no economic or healthcare utilisation benefit. Whilst this provides a signal of potential benefit at a patient-level, further controlled trials are warranted to observe causality, and to explore whether these outcomes are replicable for older people who are frail or pre-frail.

Numerous contextual factors may impact on the success or failure of social prescription programmes in frail populations. The impact of other medical comorbidities, namely dementia/cognitive impairment, and the role of informal caregivers are two key factors. Compared with non-frail participants, pre-frail and frail participants have a greater risk of developing dementia [12]. Dementia has a major impact, not just on functional capabilities and independence, but also on an individual’s ability to comply with interventions where fidelity needs to be high, such as nutritional supplements and exercise/physical activity programmes. One approach to address these issues is to prescribe interventions to a caregiving-care recipient dyad. Through such a model, the attitudes of the caregiver towards the intervention, particularly when prescribed by a non-healthcare institution, are important. Further exploration of this inter-connected relationship for this population is important to better understand the mechanisms of social prescription for frail older people.

## 5. Conclusions

The effectiveness of social prescription to prevent or delay frailty in community-dwelling older adults is unknown. This is a research priority, as social prescription is currently encouraged for this population with insufficient evidence underpinning its adoption.

## Supplementary Materials

The following are available online at https://www.mdpi.com/2308-3417/4/4/65/s1, Supplementary File S1: Search strategy presented for MEDLINE.
