# Supplementary material for: Social Prescribing Programmes to Prevent or Delay Frailty in Community-Dwelling Older Adults
Source: Geriatrics (Basel). 2019 Nov 27;4(4):65. doi: 10.3390/geriatrics4040065 (PMC6960851; doi:10.3390/geriatrics4040065)
Supplement: Supplementary file 1 [file geriatrics-04-00065-s001.pdf]

geriatrics

Supplementary File 1: Search strategy presented for MEDLINE

1. (((old or aged or geriat* or 65+ or "65") and over) or older adults or elder*).mp. [mp=title, abstract, original title, name of substance word, subject heading word, floating sub-heading word, keyword heading word, organism supplementary concept word, protocol supplementary concept word, rare disease supplementary concept word, unique identifier, synonyms]
2. (frail* or prefrail or pre-frail).mp. [mp=title, abstract, original title, name of substance word, subject heading word, floating sub-heading word, keyword heading word, organism supplementary concept word, protocol supplementary concept word, rare disease supplementary concept word, unique identifier, synonyms]
3. 1 or 2
4. (community living or community based or free living or freeliving or independent).mp. [mp=title, abstract, original title, name of substance word, subject heading word, floating sub-heading word, keyword heading word, organism supplementary concept word, protocol supplementary concept word, rare disease supplementary concept word, unique identifier, synonyms]
5. 3 and 4
6. (weight management or diet* or food club or weight loss program* or weight reduction program* or lunch club or cooking club or cooking class or cooking course).mp. [mp=title, abstract, original title, name of substance word, subject heading word, floating sub-heading word, keyword heading word, organism supplementary concept word, protocol supplementary concept word, rare disease supplementary concept word, unique identifier, synonyms]
7. (((((((((((psychosocial* or peer*) adj3 support*) or community) adj3 group*) or wellbeing) adj (service or support)) or psychoeducation* or social) adj3 (skill* or support*)) or self) adj3 help) or computer*adj3 CBT or timebank* or befriend*).mp. or (community adj3 educat*).ti,ab. or loneliness.mp. or anxiety.mp. or adult learning.mp. [mp=title, abstract, original title, name of substance word, subject heading word, floating sub-heading word, keyword heading word, organism supplementary concept word, protocol supplementary concept word, rare disease supplementary concept word, unique identifier, synonyms]
8. (physical activ* or swimming or dance classes or yoga or team sport or exercise or group activities or gym-based activities or green gym or gardening clubs or cycling or aqua-therapy or health walks or fitness*).mp. [mp=title, abstract, original title, name of substance word, subject heading word, floating sub-heading word, keyword heading word, organism supplementary concept word, protocol supplementary concept word, rare disease supplementary concept word, unique identifier, synonyms]
9. 6 or 7 or 8
10. (social prescribing or community referral or referral scheme* or well-being program* or wellbeing program* or exercise referral scheme*).mp. [mp=title, abstract, original title, name of substance word, subject heading word, floating sub-heading word, keyword heading word, organism supplementary concept word, protocol supplementary concept word, rare disease supplementary concept word, unique identifier, synonyms]
11. 9 and 10
12. 5 and 11
13. limit 12 to randomized controlled trial
